# On the Altered Specific Powers of Vaccine and Variolous Matter

**Published:** 1814-09

**Authors:** Robert Kinglake

**Affiliations:** Taunton


*For the Medical and Physical Journal.*

### *On the altered Specific Powers of Vaccine and Variolous Matter; by* Dr. Kinglake.

EVERY friend to humanity must have experienced the highest gratification in having borne full testimony to the anti-variolous power of vaccine matter. It has now had the benefit of ample trial, and has been considered and reconsidered with all the acuteness of criticism that its important pretensions merited. It has completely justified all that was affirmed of its powers by the respectable person who first introduced it to public attention. It indeed but rarely happens that commendation so fully warrants itself as that which has been justly bestowed on the preventive efficacy of vaccine against variolous infection. This proves that it was not ushered into public notice on equivocal grounds, but on the authority of correct observation of its powers; and it farther evinces that it will abide the test of all farther investigati[illegible]n that may be made concerning it. Professing then, as it unquestionably does, adequate anti-variolous power in its genuine state, it becomes an object of high importance to secure to it unimpaired, both in reality and in reputation, these inestimable properties. It is with a view to realise this advantage, that it seems to me as expedient to submit a few observations on what appears to me to be an altered or degenerated state of vaccine matter.

It has within these few years happened to me, in numerous instances, to have known the impossibility of imparting the vaccine disease by inoculation, and some cases have occurred to me in which an incomplete and an inadequate infection for the intended purpose was given. This insufficiency and these failures appear to me to be justly referable to a deteriorated riorated state of the vaccine fluid from having repeatedly undergone the vital actions exerted on it in the human system. It is reasonable to suppose that in the way of attenuation or exhaustion of the specific property of vaccine virus, it must undergo changes by being indefinitely transferred from one individual to another; and, if this actually takes place, it then ceases to be the preventive agent against a formidable disease, that has gained it so much confidence and fame. It is indeed difficult to conceive how any fluid can be so indestructably arranged as for ever to resist the decomposing influence of the chemico-animal actions to which it may be subjected, and to which vaccine matter is actually exposed. There may be a power of resistance insuring its specific arrangement during a long period in performing its preventive services against the variolous disease; but the extent of this period is imaginary, and its probable termination can only be inferred from its ceasing to act specifically when it has been employed in a protracted series of inoculations. The mass of evidence extant on the preventive efficacy of the vaccine fluid against the small-pox is most consoling, but it is not so satisfactory to be aware that, if the specific powers of the disease were not fully exerted, the security might not be as lasting as expectation; that, although a temporary insusceptibility for variolous disease might be induced, yet that it has not sufficiently identified itself with the system to be a permanent preventative. Not a year elapses but persons of limited opportunities for learning the events of vaccine inoculation hear of failures of the vaccine disease in securing against the infection of small-pox. Many of these reputed failures, it must be owned, cannot be satisfactorily verified on inquiry, yet the whole cannot be fallacious; and it would appear that instances enough have occurred fairly to impugn the infallibility of vaccine efficacy.

My opinion on this subject is, that the vaccine fluid is too long used in the human system without renovating it from the cow, which, being its genuine source, must be supposed to yield it in its most efficient specific powers. Why then not frequently resort to the animal that, by a morbid action peculiar to its nature, furnishes the desired preservative? The dairy districts are often affording natural instances of the disease; and, if this opportunity did not offer with sufficient regularity, would it not be practicable always to reserve a portion of the immediate vaccine fluid with which to infect a cow, so that it may be thus genuinely supplied as occasion might require? Does vaccination directly from the cow produce a more violent disease than when it is transferred from one subject to another? If so, the security against small small-pox would probably be proportionately great, and yet the higher degree of the disease would not be accompanied with any calculable risk of life. The disease, as contracted from the infected cow, is I believe usually attended with general eruptions over the skin; but to my knowledge it is not on record that it has in any instance proved mortal. This being the case, it behoves, in my judgment, the advocates for the real benefit and permanent reputation of vaccine inoculation, to go to the cow very often for a fresh supply of genuine fluid, in which could not fail to be found those unaltered specific powers on which its preventive efficacy against small-pox depends. Either recourse should be had to the cow in every instance, or, consistently with the motive for doing so, the same matter should not be re-inoculated perhaps more than six times. To that extent no diminution of power may probably be sustained; but this is a question that can only be absolutely set at rest by uniformly recurring to the animal that has originated the morbid fluid.

That the vaccine fluid undergoes some change by long-continued inoculation, derives support from the notorious fact that, since the comparative discontinuance of inoculation for the small-pox, the variolous fluid, from being almost entirely furnished by natural cases, is found on being employed in inoculation to induce a much more virulent disease than when the frequency of the practice caused it to be transferred from one inoculated subject to another, by which the matter was probably rendered more mild than when it resulted from the natural form of the disease, so as to impart to the inoculated small-pox a morbid character almost free from calculable risk. This is a reason why a partial indulgence of inoculation for the small-pox becomes a public evil, by subjecting to hazard the lives of those who under a more general practice would have to undergo a comparatively mild and safe form of the disease. The prohibition of variolous inoculation, therefore, by a suitable legal enactment, would be imposing a wise, humane, and beneficial restraint, which, aided by the anti-variolous efficacy of the vaccine disease, would afford a reasonable prospect at no distant time of altogether exterminating the small-pox. Inoculation for the small-pox keeps the disease always in action, and by subjecting persons to casual infection, has proved rather a scourge than a benefit to mankind, in as far as the deaths resulting from the frequency of the natural disease, owing to inoculation, are held to have exceeded the average amount of mortality from that disease occurring for any given period previously to the introduction of variolous inoculation.

The extinction of the small-pox is an object worthy of the best best endeavours of humanity, and assisted by the most efficient mode of applying the preventive powers of the vaccine fluid, it may be justly regarded as an event fairly within the scope of rational expectation.

*Taunton,* ROBERT KINGLAKE.

*July* 25, 1814.

